# Precancerous microenvironment: A signalling perspective

**DOI:** 10.1016/j.ceb.2025.102611

**Published:** 2026-02

**Authors:** Xiao Qin

**Affiliations:** MRC Translational Immune Discovery Unit, MRC Weatherall Institute of Molecular Medicine, University of Oxford, Oxford, OX3 9DS, UK

## Abstract

The progression from healthy tissue to malignancy involves a critical precancerous stage marked by cellular lesions with aberrant molecular and phenotypic characteristics. The fate of these lesions is shaped not only by cell-intrinsic alterations but also by the precancerous microenvironment (PME), an ecosystem of epithelial, stromal and immune cells embedded within the extracellular matrix. Focusing on epithelial precancers, this review first defines the metastable state and signalling networks that distinguish precancer from homeostasis and cancer. It then examines the models and technologies used to investigate PME signalling across spatial–temporal dimensions, followed by an integrated overview of how PME components collectively shape lesion trajectories. Finally, it outlines the outstanding questions and research priorities needed to advance mechanistic insight and realise the translational potential of PME-targeted interventions.

## Introduction

**Figure 1:**
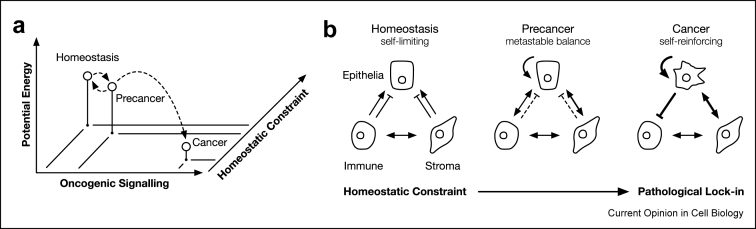
**Precancer as a metastable state maintained by competing signalling networks (a)** Energy landscape model of tissue transformation. Precancerous lesions occupy a metastable state – a local energy minimum distinct from both homeostasis and cancer. The balance between oncogenic signalling and homeostatic constraints shapes the potential energy landscape and determines state stability. Depending on this balance, precancerous lesions can regress, persist, or progress. **(b)** Signalling network architecture underlying the energy landscape. In homeostasis, epithelial proliferation is restricted by reciprocal negative–feedback interactions among epithelial, stromal, and immune compartments. In precancer, these constraining signals weaken (dashed arrows), while epithelial cells engage in bidirectional signalling that provides a competitive growth advantage. In cancer, transformed epithelial cells establish autocrine loops and reprogramme stromal cells, creating self-reinforcing positive feedback and pathological lock-in.

Despite significant advances in diagnosis and treatment, cancer remains a leading cause of morbidity and mortality worldwide. A critical window for intervention exists in the early stages of neoplastic development, where precancerous lesions remain localised and potentially reversible, their progression constrained by tissue architecture and microenvironmental cues (Figure 1a).

Precancerous lesions represent a continuum of histological and molecular abnormalities marking the transition from normal tissue homeostasis towards malignant transformation [1]. This review examines the signalling dynamics that govern epithelial precancer, where the precancerous microenvironment (PME) comprises genetically and epigenetically altered epithelial cells, stromal and immune populations, and an evolving extracellular matrix (ECM) [2]. These components communicate through signalling networks that integrate cell-intrinsic and microenvironmental cues, ultimately dictating whether a lesion regresses, remains dormant, or progresses towards malignancy.

A defining feature of the PME is that it operates within a homeostatic tissue framework, where cellular crosstalk and ECM interactions are regulated by negative feedback loops and mechanical constraints (Figure 1b). As lesions evolve, these homeostatic controls become progressively destabilised. A critical inflection point arises when feedback mechanisms fail to restrain aberrant signalling, giving rise to self-reinforcing loops that establish a new, pathological equilibrium – the hallmark of the tumour microenvironment (TME). Understanding the principles that govern the buffering capacity of normal tissues, and the conditions under which such constraints collapse, is essential for elucidating the mechanisms of precancer progression and informing early detection and prevention strategies.

## Models and methods for studying the PME

**Figure 2:**
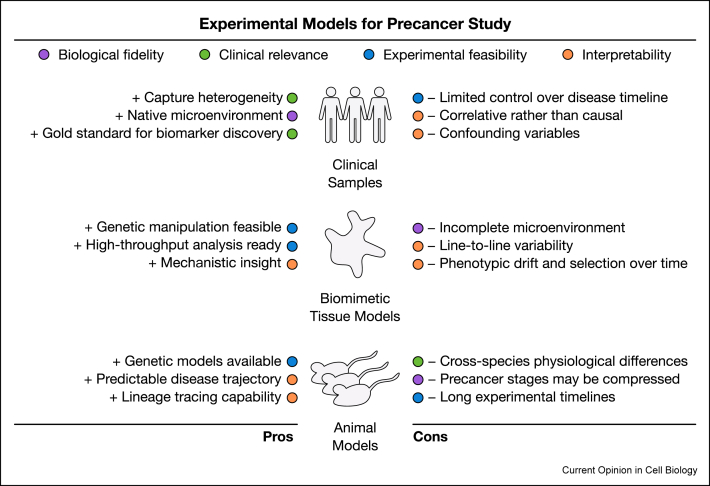
**Systematic comparison of experimental approaches for studying precancer.** Clinical samples provide unmatched biological fidelity and clinical relevance but offer limited experimental control and primarily correlative insight. Biomimetic tissue models enable mechanistic dissection and high-throughput analysis, though they lack full microenvironmental complexity and are susceptible to line-to-line variability. Animal models preserve native tissue architecture and allow longitudinal tracking but face cross-species differences and often compress the prolonged metastable precancerous phase characteristic of human disease. Complementary use of multiple model systems will yield the most comprehensive understanding of precancerous signalling.

The PME is an active driver of malignant transformation. Understanding how it arises from homeostatic tissue and evolves towards malignancy requires experimental systems that capture its spatial–temporal complexity while remaining mechanistically tractable (Figure 2).

Patient samples from screening programmes offer the most clinically relevant view of precancerous lesions. However, since progression to overt malignancy can span decades, capturing the full trajectory within a single cohort remains challenging. Non-invasive approaches such as liquid biopsy and advanced imaging have improved early cancer detection, yet their sensitivity for subtle, macroscopically indistinct precancerous states remains limited [3]. These limitations can be partly addressed through carefully curated cancer cohorts that encompass samples spanning multiple stages of tumourigenesis within the same patients, thereby capturing the continuum of malignant transformation and illuminating the dynamic, stage-specific roles of PME components [4,5]. Genetically predisposed cohorts – such as individuals with familial adenomatous polyposis, Lynch syndrome, and Li-Fraumeni syndrome – offer strategic opportunities to interrogate early PME dynamics owing to their relatively well-defined pathological milestones [6]. Although findings from such cohorts may not fully generalise to sporadic cancers, they nonetheless provide a valuable framework for multi-omic dissection of both cell-intrinsic and -extrinsic mechanisms driving tumour development [7].

Given the limitations of studying precancer biology directly from clinical samples, a spectrum of *in vitro* models has been developed to capture the early mechanistic steps of tumourigenesis. These include genetically engineered organoid models recapitulating key steps of tumourigenesis [8,9] and long-term cultures that model tissue transformation over time [10,11]. Building on conventional organoid systems, advanced biomimetic platforms such as organ-on-chip technologies [12] and patient-derived explants [13], seek to reproduce the structural and cellular complexity of the PME while enabling controlled functional interrogation. Although these platforms have not yet been widely applied to precancer studies, they hold considerable promise to serve as functional biopsies for dissecting and targeting heterocellular signalling networks within the PME.

To complement patient-derived models and delineate the mechanistic drivers of precancer evolution, autochthonous animal models remain indispensable [14]. Genetically engineered mouse models (GEMMs) enable temporally controlled, tissue-specific interrogation of stepwise tumour initiation [15]. When coupled with genome-engineering strategies such as lineage tracing [16] and functional signalling reporters [17], GEMMs allow longitudinal tracking of cellular and molecular transitions during precancer evolution, providing mechanistic insights unattainable from observational human studies [18]. Although GEMMs do not fully capture the genetic heterogeneity or immune complexity of human disease, advances in patient-derived xenografts and humanised mouse models offer promising routes to bridging these gaps and yielding increasingly physiologically relevant representations of the PME [19].

As powerful as these models are, their impact ultimately depends on analytical pipelines capable of resolving cellular and temporal dynamics at scale. Single-cell and spatial profiling techniques now enable multi-modal interrogation of the PME, revealing the structural, cellular, and molecular changes that accompany the transition from precancer to cancer [20]. Crucially, advanced lineage tracing and molecular recording methodologies have made the temporal dimension – arguably the most informative axis in cancer development – experimentally tractable [21,22]. This integrated spatial–temporal resolution enables unprecedented precision in tracking progressing clones and delineating their evolutionary trajectories [23,24]. Crucially, interpreting these complex datasets allows the generation of robust, data-driven hypotheses that can be experimentally validated – an iterative cycle that underpins modern systems biology, harmonising computational analysis with laboratory experimentation and clinical observation. The integration of high-dimensional profiling, computational modelling, and functional validation moves beyond descriptive correlation towards identifying causal signalling nodes that determine the fate of precancerous lesions.

## The PME signalling landscape

**Figure 3:**
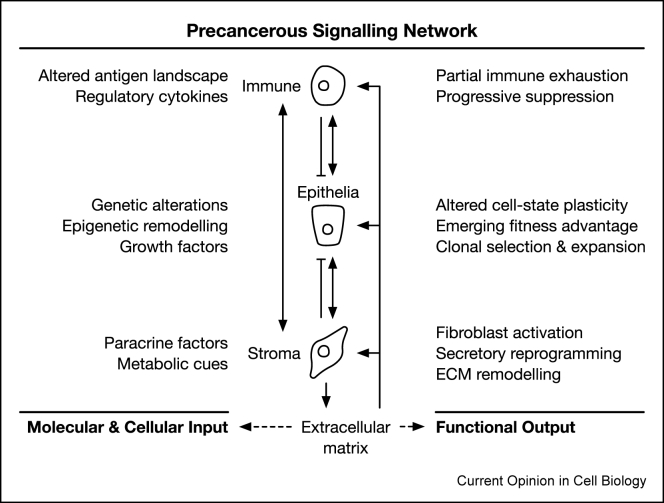
**Precancerous Signalling Network.** Molecular and cellular inputs act on immune, epithelial, and stromal compartments to generate functional outputs that collectively shape the PME. Solid arrows denote direct intercellular signalling; dashed arrows represent ECM-mediated effects. Together, these interactions form a dynamic, reciprocal network that imposes competing selective pressures and sustains the metastable precancer state.

The PME is shaped by intricate interactions among epithelial, stromal, and immune cells within the ECM [2]. Despite tissue-specific differences, most models of precancer evolution converge on three core components: epithelial-intrinsic alterations (somatic mutations, chromatin instability, epigenetic dysregulation), stromal remodelling (fibroblast activation, ECM degradation, aberrant vascular architecture), and immune modulation (a shift from surveillance to immunosuppressive phenotypes) [1]. The signalling interplay across these axes is both hierarchical and reciprocal: epithelial alterations provide early proliferative and survival advantages; stromal remodelling redefines the molecular and mechanical landscape; and immune modulation ultimately determines whether precancerous cells are eliminated or allowed to persist. Epithelial clones that generate robust pro-survival signals, co-opt the stroma, and evade immune surveillance are selected for expansion, driving precancer towards malignancy. The following section outlines how signalling within and between these compartments defines the PME and determines lesion fate (Figure 3).

### Epithelial focus

Epithelial transformation is widely regarded as the initiating event in tumourigenesis. Somatic mutations in canonical pathways such as MAPK, PI3K, WNT, KRAS, and P53 confer proliferative and survival advantages [1], yet emerging evidence suggests that these genetic alterations alone are insufficient for overt malignancy [25,26]. Epigenetic aberrations, including enhancer reprogramming [26] and altered DNA methylation [27,28], further promote transformation by enhancing epithelial plasticity. These plastic states are characterised by loss of stable homeostatic identities, acquisition of progenitor-like regenerative phenotypes, and ambiguous lineage specification, collectively fostering a heterogeneous cellular landscape permissive to malignant progression [9,29,30].

The adoption of a hyperplastic, regenerative fate by precancerous epithelial cells elicits two interconnected cell-extrinsic programmes: secretory reprogramming [31] and metabolic rewiring [32]. In pancreatic neoplasia, for example, Kras^G12D^ mutant cells activate IL-33 signalling, creating a pro-tumourigenic feedback loop with Th2 cytokine-producing regulatory T cells (Tregs) and innate lymphoid cells that drives reciprocal oncogenic tissue remodelling [15]. In parallel, precancerous epithelial cells increase aerobic glycolysis (the Warburg effect), supporting anabolic growth and survival under stress [33,34]. Enhanced glycolytic flux not only fuels epithelial biosynthesis but also generates signalling metabolites that modulate adjacent stromal and immune populations, fostering a permissive microenvironment that, in turn, sustains epithelial transformation [33].

### Stromal focus

In response to epithelial secretory and metabolic reprogramming, the stromal compartment comprising fibroblasts, endothelial cells, pericytes, and mesenchymal stem cells [1] becomes activated and collectively supports, constrains, or remodels the evolving lesion. Although fibroblast functions are better characterised in established cancers [35], those in low-grade precancerous lesions already acquire heterogeneous phenotypes reminiscent of cancer-associated fibroblasts (CAFs), engaging in context-dependent paracrine signalling, metabolic reprogramming, and ECM remodelling [36].

Reciprocal epithelial–stromal signalling continuously remodels the PME throughout tumourigenesis. For example, Kras^G12D^ epithelial cells in precancerous pancreatic lesions activate fibroblasts to secrete pro-inflammatory cytokines (CXCL1, IL-33, IL-6), which promote pro-tumourigenic macrophage polarisation whilst impairing tissue repair [37]. Activated fibroblasts further reshape immune function via fibroinflammatory signalling that both promotes tumourigenesis [38] and imposes immunosuppression [39]. Senescent fibroblasts contribute via the senescence-associated secretory phenotype (SASP) characterised by persistent secretion of IL-6, IL-8, and matrix-remodelling enzymes, which amplifies paracrine inflammation and epithelial plasticity, promoting epithelial proliferation, migration, and invasion while dampening immune surveillance [40]. Fibroblasts can also adopt glycolytic metabolism, releasing lactate and pyruvate that neighbouring cancer cells utilise for biosynthesis, establishing a metabolically symbiotic microenvironment [41].

Beyond paracrine and metabolic signalling, stromal cells remodel the physical and mechanical architecture of the PME through ECM deposition and modification [42]. ECM proteins such as collagen XVIII and fibronectin 1 accumulate in chronic inflammation, creating a stiffened, growth-promoting matrix that parallels early tumour development [43]. Conversely, matrix metalloproteinases (MMPs) degrade ECM components, loosening the microenvironment to facilitate lesion expansion [44,45].

Critically, stromal function evolves dynamically with lesion progression. Early desmoplastic responses can be tumour-suppressive, physically containing nascent outgrowths [4,46]. As disease progresses, however, emerging clones can co-opt these pathways, transforming protective fibrosis into a pro-invasive niche [36]. TGF-β signalling exemplifies this duality, shifting from growth-inhibitory to pro-invasive and immunosuppressive roles as disease advances [47]. Understanding when and how such signals switch function remains a fundamental open question in PME biology.

### Immune focus

The interaction between immune cells and neoplastic outgrowth represents another critical axis determining precancerous lesion fate, evolving from active surveillance to immunosuppression [1].

Initially, the immune system recognises and eliminates transformed cells via antigen-presenting cells, primarily dendritic cells, which capture neoantigens and prime cytotoxic T cell responses [48]. Although cytotoxic T cells can effectively constrain clonal expansion, chronic antigen exposure and an immunosuppressive cytokine milieu drive T cell exhaustion [49]. This is reinforced by upregulation of immune checkpoint ligands on epithelial or myeloid cells, ultimately establishing an immunosuppressive niche [36]. In parallel, tissue-resident macrophages contribute to a pro-tumourigenic microenvironment through chronic inflammation and niche remodelling, as demonstrated in precancerous lung adenocarcinoma (LUAD) [50,51] and liver cancer [52] precursors.

Beyond T cell exhaustion and checkpoint signalling, the PME progressively assembles a broader suppressive network that reinforces immune evasion. First, it recruits and expands dedicated suppressor populations, including Tregs and myeloid-derived suppressor cells [53]. Second, macrophages transition from pro-inflammatory to anti-inflammatory, pro-repair states, with TIM-3^+^ macrophages emerging as key suppressors in LUAD precursors [51]. Third, metabolic competition restricts effector T cell access of essential nutrients, thereby limiting their cytotoxic function [54]. This multifaceted suppressive network represents a pivotal transition in precancer evolution, establishing an immune-privileged site that enables unchecked clonal expansion and progression.

In summary, the signalling landscape of the PME emerges through tripartite crosstalk that transitions from self-contained conflict to metastable symbiosis. Early epithelial alterations trigger a cascade of reciprocal signals with stromal cells, driving their activation and functional diversification. Activated stroma provides pro-survival cues, metabolic support, and remodelled ECM that sustain epithelial proliferation and plasticity. Ultimately, the combined epithelial–stromal signalling output systematically undermines anti-tumour immunity, establishing an immunosuppressive niche that facilitates malignant progression.

## Concluding remarks and future perspectives

Recent advances in precancer models and high-dimensional profiling have substantially deepened our understanding of PME signalling, yet several critical questions remain unanswered. A primary challenge is to predict which lesions will progress to invasive cancer; the signalling profiles of high-risk lesions are likely to be particularly informative in this regard. Longitudinal, multi-omic profiling of large, clinically annotated patient cohorts will be essential for identifying robust biomarkers of malignant potential. In parallel, advances in artificial intelligence are beginning to integrate complex, multidimensional data with clinical outcomes, creating new opportunities for predictive modelling of lesion evolution [55]. Another outstanding question concerns the continuity and divergence of molecular and cellular programmes during the transition from precancer to malignancy: to what extent are early alterations preserved, remodelled, or superseded? Longitudinal studies facilitated by lineage tracing technologies [56] should clarify which pathways remain “locked in” during the process – a critical insight for uncovering therapeutic vulnerabilities relevant to both prevention and early intervention.

Resolving these questions holds substantial translational potential. A deeper mechanistic understanding of PME signalling could inform cancer interception strategies that extend beyond targeting transformed epithelial cells to include reprogramming stromal responses, disrupting metabolic symbiosis, or reversing immunosuppressive niches through selective modulation of precancer-specific checkpoints. Equally, defining the molecular hallmarks of PME signalling could transform early detection. Circulating tumour DNA bearing precancer-associated methylation signatures offers a tractable route, given that epigenetic alterations arise early in lesion evolution [27,28]. Complementary approaches may involve detection of stromal- or immune-derived circulating factors, although these require additional clinical validation. Integrating such PME-derived biomarkers into non-invasive screening platforms could enable identification of high-risk lesions in asymptomatic individuals, providing a feasible path towards scalable population-level surveillance and early interception [3].

Ultimately, integrating molecular risk stratification with mechanistic insight into PME signalling should enable predictive models and therapeutic strategies tailored to the earliest – and most tractable – stages of tumourigenesis, bridging cancer prevention, early detection, and precision intervention.

## Declaration of competing interest

There are no competing interests to disclose.

## Data Availability

No data was used for the research described in the article.
